# Editorial: Reviews in Psychiatry 2022: Schizophrenia

**DOI:** 10.3389/fpsyt.2023.1237676

**Published:** 2023-07-11

**Authors:** Massimo Tusconi, Teresa Sanchez-Gutierrez

**Affiliations:** ^1^Section of Psychiatry, Department of Medical Sciences and Public Health, University of Cagliari, Cagliari, Italy; ^2^Faculty of Health Science, Universidad Internacional de La Rioja (UNIR), Logroño, La Rioja, Spain

**Keywords:** reviews, Schizophrenia, biomarkers, therapeutic targets, systematic reviews, RNA, altered protein levels, psychosocial functioning

The reviews aim to collect and analyze in an all-encompassing light article separately published in the literature to provide a higher-order view that allows a broader assessment of the topic described, all in a thorough and unbiased manner (1). The previous view based only on certain pathology aspects can no longer be considered sufficient to achieve an optimal outcome. Integrating microscopic and macrobiological aspects, identifying biomarkers, and the need for integrated care that includes all aspects currently considered in the scientific literature becomes essential in the understanding of psychiatric disorders (2).

Therefore, an overview of qualitative and quantitative data could lead to a further and deeper understanding of the kind, molecular, and clinical mechanisms underlying the spectrum of Schizophrenia (3).

In this context, the goal of our Research Topic was to highlight the most recent advances on these fields, equally reinforcing important new emerging aspects concerning new research and new directions. The results of the reviews analyzed allowed for reflection on multiple aspects of mental pathology and for careful consideration of which aspects are crucial to direct scientific advancement in the future.

Concerning the clinical aspects of the condition, it appears that the result of the action of befriending on patients diagnosed with Schizophrenia and associated symptoms is currently greatly underestimated. The review by Farcas et al. shows that guidelines have gradually tended to incorporate psychotherapeutics and social integration into treatment in addition to pharmacotherapy aiming to improve quality of life of people with Schizophrenia. In particular, they have noted befriending as a possibility to increase the strengthening of relationships and interactions at the social level, hypothesizing its complementary implementation in patients with Schizophrenia, particularly in situations where a CBT is not accessible. In their review paper on Cognitive Remediation Training, Lu et al. highlight how psychoeducation, in combination with cognitive remediation training and social skills training, can effectively enhance the psychosocial functioning in schizophrenic spectrum disorders, especially in patients with the first episode.

In a systematic review conducted across a broad spectrum of risk factors, Reilly et al. found no strong evidence for an marked correspondence between negative impact life events and the occurrence of postpartum psychosis. However, there are data indicating that genetics contributes to the development of postpartum psychosis. It also emerges how ascertaining what factors contribute to pathological relapse is paramount to reducing long-term negative outcomes, especially because more than half of women with postpartum psychosis will go on to experience subsequent episodes even at a distance in time, with a significant risk of suicide.

Delving into the analysis of the reviews presented on the more biological and molecular aspects, one can see pathological elements underlying multiple aspects of the pathology. In particular, Rawas et al. findings revealed how there are studies performed in patients with Schizophrenia focusing on the expression of CaMKII in the frontal cerebral cortex post-mortem.

In addition, Anserey highlights a precise alteration of cellular pathology underlying the decreased flush response in his review. First, it is inferred that the decrease in flushing is caused not only by the effect of vasodilators, but also through altered protein expression, action and inflammatory balance. Concerning altered protein levels in the GPR109A-COX-prostaglandin pathways are included enzymes, cPLA2 and COX-2, and also membrane phospholipids, GPR109A, and prostaglandins, with receptors and derivatives such as EP2, EP4, 15d-PGJ2, PGD2, PGE2, DP1, and PPARy.

Furthermore, Georgiou et al. illustrate how out of a total of 17 articles on methodological designs, 3 found a clear improvement in cognition after the reduction of anticholinergic therapy. In addition, 13 studies have shown an association between high anticholinergic level and cognitive impairment with assessment of composite neurocognitive scores and individual cognitive domains. In particular, these effects were shown on memory, learning, processing speed and execution.

Wu et al. have emphasized the critical role of some dysregulated lncRNAs in the pathogenesis of Schizophrenia. The biological mechanisms of lncRNAs in psychosis still remain in the exploratory stage, and consequently, the development of new diagnostic pathways and therapy in this regard are yet to be developed.

Finally, Zhang et al. focus on the therapeutic aspects of psychotic pathology, specifically examining Adjunctive Magnetic Seizure Therapy (MST) for Schizophrenia, revealing how additional MST has been shown to reduce major symptoms, evaluating the effects through PANSS and BPRS; they also found no adverse effects on the neurocognition. As reported in the studies reviewed, the response rate of the additional STD in Schizophrenia was between 37.5% and 50%, while that for ECT was 74%.

In summary, this Research Topic has shown that the overall analysis of data and synthesis of findings found in studies of different types and methodologies emphasizes aspects not apparent from the evaluation of individual papers, posing as an essential aspect of research, especially in those areas where knowledge is not fully harmonized with each other for multiple reasons. Identifying new aspects using these analytical methodology could promote new strands of clinical and basic research, and lead medical science in promoting diagnostic and therapeutic choices that can comprehensively improve patient outcomes and quality of life.

## Author contributions

All authors listed have made a substantial, direct, and intellectual contribution to the work and approved it for publication.
